# Angiofibroma of the external auditory canal: a rare case with literature review

**DOI:** 10.1093/jscr/rjac117

**Published:** 2022-04-03

**Authors:** Khalid Bouhafs, Azeddine Lachkar, Tayeb Bouamama, Achraf Miry, Drissia Benfadil, Mohammed Rachid Ghailan

**Affiliations:** Otorhinolaryngology and Head and Neck Surgery Department, Mohammed VI University Hospital, Oujda, Morocco; Faculty of Medicine and Pharmacy, Mohammed Ist University, Oujda, Morocco; Otorhinolaryngology and Head and Neck Surgery Department, Mohammed VI University Hospital, Oujda, Morocco; Faculty of Medicine and Pharmacy, Mohammed Ist University, Oujda, Morocco; Faculty of Medicine and Pharmacy, Mohammed Ist University, Oujda, Morocco; Department of Radiology, Mohammed VI University Hospital, Oujda, Morocco; Faculty of Medicine and Pharmacy, Mohammed Ist University, Oujda, Morocco; Department of Anatomic Pathology, Mohammed VI University Hospital, Oujda, Morocco; Otorhinolaryngology and Head and Neck Surgery Department, Mohammed VI University Hospital, Oujda, Morocco; Faculty of Medicine and Pharmacy, Mohammed Ist University, Oujda, Morocco; Otorhinolaryngology and Head and Neck Surgery Department, Mohammed VI University Hospital, Oujda, Morocco; Faculty of Medicine and Pharmacy, Mohammed Ist University, Oujda, Morocco

## Abstract

Angiofibromas represent <1% of all head and neck tumors and occur primarily in the nasopharynx. Extra-nasopharyngeal angiofibromas are rarer. Remarkably, only a case of external auditory canal location has been reported. We present a case of an angiofibroma in this unique location in a female who presented with fullness of right ear and hypoacusis for 6 months. The clinical examination found a mass in the right external auditory canal attached to the posterosuperior wall. Preoperative audiometry revealed average right conductive hearing loss of 37.5 dB. A computed tomography scan revealed a mass on the right external auditory canal. Surgical resection of the mass was performed and the histopathological assessment confirmed the diagnosis. Post-operative audiometry showed an improvement in hearing function. There was no recurrence after 5 years. The prognosis of these tumors is good after total bloc resection.

## INTRODUCTION

Angiofibromas account for <1% of all head and neck tumors and occur primarily in the nasopharynx (PNA) and mainly affect adolescent males [1–3]. Extra-nasopharyngeal angiofibromas (ENPA) are even rarer, with <80 cases reported in the literature and with only 1 case originating from the external auditory canal [1, 2]. In this case report, we discuss the first case of this entity in a woman from the Moroccan Oriental region.

## CASE PRESENTATION

**Figure 1:**
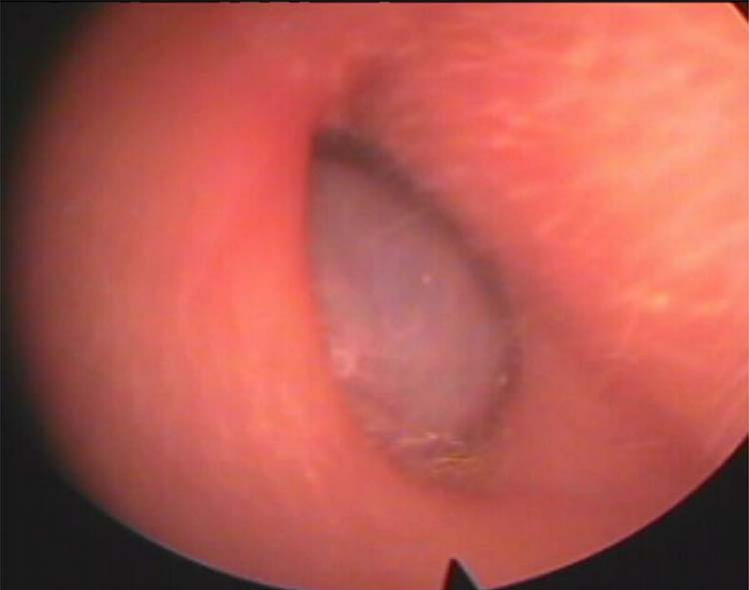
Mass of the right external auditory canal.

**Figure 2:**
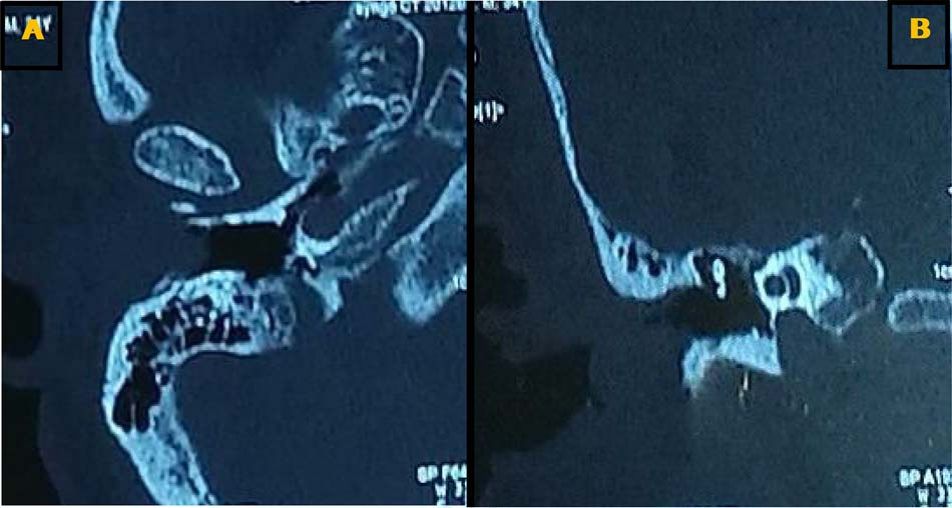
Axial (**A**) and coronal (**B**) CT scans showing a tissue lesion process of the external auditory canal with erosion of the eardrum and filling of a few mastoid cells.

Our patient was a 40-year-old female, with unremarkable disease history, who presented with a fullness of the right ear for 6 months which was associated with progressive hypoacusis and intermittent ipsilateral tinnitus. The clinical examination showed a mass in the right external auditory canal, attached to the posterosuperior wall, which was smooth, pink, firm, mobile, well limited and hemorrhagic on contact (Fig. 1). Weber and Rinne tuning fork tests were negative and right-lateralized, respectively. Preoperative pure tone audiometry revealed an average right conductive hearing loss of 37.5 dB. Imaging based on computed tomography (CT) scan showed a mass occupying the outer two-thirds of the right external auditory canal, measuring 17 mm, well limited, with regular contours. The tissue radio density measured was 61 HU with erosion of the middle ear (Fig. 2).

**Figure 3:**
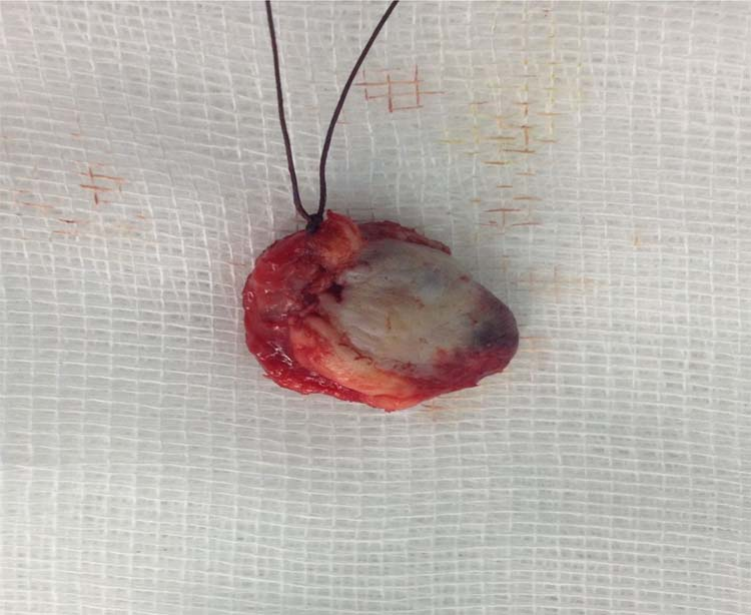
Surgically resected specimen.

**Figure 4:**
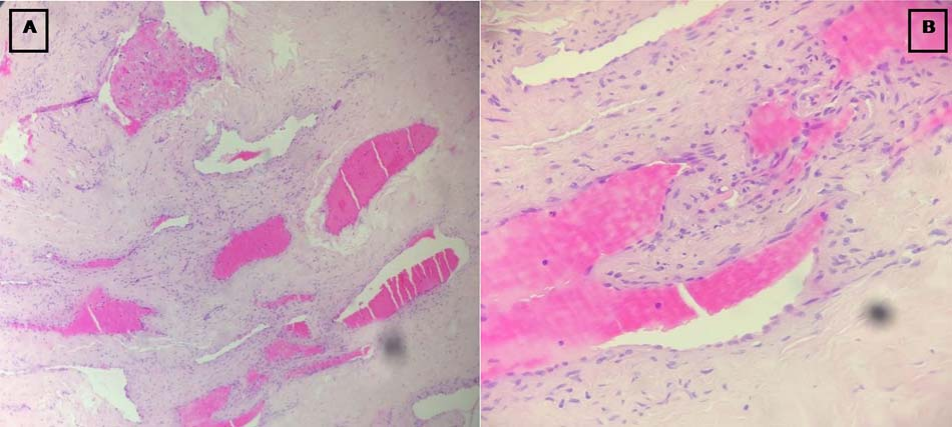
Images showing the fibroblastic (**A**) and vascular (**B**) components of the tumor.

A surgical resection of the mass was performed via a retro-auricular incision. The mass was resected with the eardrum being intact (Fig. 3). Histological examination was performed by a qualified pathologist, and angiofibroma was retained as the diagnosis. It was composed by a benign tumor proliferation with two cellular features, a fibroblastic component of spindle-shaped cells with oval- or spindle-shaped nuclei, bathed within multiple collagen bundles and a vascular component composed by numerous small vessels dispersed within the fibroblast proliferation, without signs of malignancy (Fig. 4). Post-operative examination by audiometry revealed a hearing gain of 25.5 dB. To date, there is no recurrence after 5 years of follow-up.

## DISCUSSION

Angiofibromas are benign fibrovascular tumors with thin-walled and irregularly shaped vessels embedded in a fibrous or cartilaginous stroma containing many spindle-shaped or stellate cells [2]. There are currently two entities, PNA and ENPA. They can be distinguished by their anatomic locations. Few published cases of ENPA were also reported in the maxillary sinus, the ethmoid sinus, the nasal septum, the nasal cavity, the sphenoid sinus, the larynx, the cheek, the tonsils, the oral cavity, the retromolar region and the conjunctiva [1–3].

Despite being histologically similar, there are remarkable differences between PNA and ENPA, which support their classification as clinically distinct entities [1–3]. For PNA, the diagnosis is often easy due to its clinical epidemiology, symptomatology, occurrence—mainly in adolescent males—and also its unique imaging features [2]. However, the diagnosis of ENPA can sometimes be missed or delayed as in the case of our patient because of the few and variable clinical signs related to their location [1–3]. CT and magnetic resonance imaging are important to show the location, size, extent of the tumor and its relationship with the surrounding tissues. Carotid or selective angiography can also be used to demonstrate tumor vascularization and to allow preoperative interventional embolization to reduce bleeding during operation [2]. Preoperative NPA biopsy is not recommended because of the high risk of bleeding. However, this is rarely seen in ENPA. Moreover, the less aggressive presentation and growth of ENPA are associated with good outcomes and rare recurrences after surgical resection [1–3]. During the diagnosis of these entities, several differential diagnoses may be proposed and this includes angiofibrolipomas and solitary fibrous tumors (SFTs), vascular tumors, infected polyps, myxomas, fibromyxoid sarcomas and liposarcomas [1–3]. As suggested by its name, angiofibrolipomas are a mixture of mature adipocytes, vascular and collagenous connective tissues. These lesions are not encapsulated but can be histologically distinguished from the surrounding tissues. Moreover, they also exhibit low to moderate cellularity and are not clinically aggressive [4]. The differential diagnoses include cutaneous angiolipoleiomyoma (also called angiomyolipoma), a reported lesion in the earlobe [5, 6]. Angiomyolipomas are frequently associated with Bourneville tuberous sclerosis. Positive Masson’s trichrome staining for collagen or negative immunostaining for smooth muscle actin are key markers to differentiate an angiofibrolipoma from an angiolipoleiomyoma [7]. On the other hand, angiofibrolipomas are thought to be hamartomas (polyclonal) as opposed to true neoplasms (monoclonal) [4]. Other tumors can also be proposed in the differential diagnosis, such as SFTs. These tumors are of mesenchymal origin and occur in various regions of the body. SFTs are composed of spindle- or oval-shaped cells with collagen stroma and various amounts of branching vessels. Sometimes, they show a ``deer horne-like'' structure. By their domination of giant cells, SFTs were previously known as giant cell angiofibroma (GCA). The scattered multinucleated giant cells often lining the pseudovascular space are typical findings of this rare tumor [8, 9]. GCAs were first described as a rare orbital tumor in 1995 by Dei Tos *et al*. [10]. However, a wider distribution of this rare tumor has been reported in other anatomical locations, such as the back, hip, retroperitoneum, mediastinum, vulva, groin and axillary soft tissues [9, 11]. Conventional SFTs of the external auditory canal are extremely rare and only two cases at this site have been reported elsewhere [12, 13]. Otalgia can be primary or secondary [14]. Clinical doctors should approach the ear in a more complete fashion and not only as a single entity. Nerve pathways may lead to a variety of symptoms.

## CONCLUSION

Angiofibromas are very rare in the head and neck regions. Their location in the external auditory canal remains even rarer. The definitive diagnosis requires a histological assessment of the surgical specimen after total excision. Being benign, these tumors are of good prognosis. Biopsy without a prior imaging is strongly contra-indicated due to the risk of bleeding.

## Data Availability

The patient’s data are available upon reasonable request to the corresponding author.
